# Review and Modification of Entropy Modeling for Steric Effects in the Poisson-Boltzmann Equation

**DOI:** 10.3390/e22060632

**Published:** 2020-06-08

**Authors:** Tzyy-Leng Horng

**Affiliations:** Department of Applied Mathematics, Feng Chia University, Taichung 40724, Taiwan; tlhorng@fcu.edu.tw

**Keywords:** steric effect, Poisson-Boltzmann model, Bikerman model, entropy, specific ion size

## Abstract

The classical Poisson-Boltzmann model can only work when ion concentrations are very dilute, which often does not match the experimental conditions. Researchers have been working on the modification of the model to include the steric effect of ions, which is non-negligible when the ion concentrations are not dilute. Generally the steric effect was modeled to correct the Helmholtz free energy either through its internal energy or entropy, and an overview is given here. The Bikerman model, based on adding solvent entropy to the free energy through the concept of volume exclusion, is a rather popular steric-effect model nowadays. However, ion sizes are treated as identical in the Bikerman model, making an extension of the Bikerman model to include specific ion sizes desirable. Directly replacing the ions of non-specific size by specific ones in the model seems natural and has been accepted by many researchers in this field. However, this straightforward modification does not have a free energy formula to support it. Here modifications of the Bikerman model to include specific ion sizes have been developed iteratively, and such a model is achieved with a guarantee that: (1) it can approach Boltzmann distribution at diluteness; (2) it can reach saturation limit as the reciprocal of specific ion size under extreme electrostatic conditions; (3) its entropy can be derived by mean-field lattice gas model.

## 1. Introduction

One of the major limitations of the Poisson-Boltzmann (PB) and Poisson-Nernst-Planck (PNP) models is the assumption of point-like ions without considering their sizes. These models based on mean field theories work well for dilute electrolytes, but break down when the concentration is high and ions are crowded in it. A high concentration would generally cause steric repulsions and additional electrostatic correlations among ions, that cannot be described by classical PB/PNP models [1]. For example, the concentration of counter-ions, predicted by PB, can be unrealistically high near the electrode surface, when the electrode voltage is large. Another example occurs at the selectivity filter in a potassium channel, where potassium ions are strongly attracted into this extremely narrow filter by the strong negative charges of oxygens on the backbone of the filter. Employing classical PB/PNP models would overestimate the density of potassium inside the filter and give incorrect channel current predictions. Therefore, many researchers have worked on the modification of PB/PNP to include the steric effect of ions.

Steric effect has long been approached in modeling by modifying either the internal energy or entropy in the Helmholtz free energy. Through internal energy, the steric effect has been featured as excess hard-sphere energy either by density functional theory (DFT) [2,3] or Lennard-Jones potential [4]. These energies were all formulated using non-local potentials and cause the resultant modified PB/PNP to produce a series of complicated integro-differential equations, which are hard to compute in higher dimensions. For practical implementations, localization of hard-sphere potential and simplifying integro-differential equations into pure differential equations has been conducted in [5,6,7,8] for DFT and [9] for Lennard-Jones potential. 

Through an entropy approach, Bikerman modified the classical Boltzmann distribution by adjusting bulk and local ion concentrations via the excluded volume concept [10]. Borukhov et al. [11] rigorously derived the same formula independently by adding solvent entropy through excluded volume into the Helmholtz free energy. Although the localized hard-sphere model-based DFT [5,6,7,8] also captures this solvent entropy as one of the terms accounting for excess hard-sphere chemical potential, the Bikerman model [10,11] has been a more popular steric model due to its easiness of application and qualitatively good agreement with experiments [12,13,14,15]. 

In order to obtain the potential and further derive a neat modified PB equation from free energy, Borukhov et al. [11] treated all ions as having identical size, which has been long criticized for neglecting specific ion sizes. Researchers have tried to address this shortcoming with specific ion sizes, and many of them simply extended original Bikerman model by replacing the identical ion size with specific ones without any rigorous justification. Although the resultant model has a better agreement with experiments than the original Bikerman model [16,17,18], it does not have a Helmholtz free energy to support it. Here modifications of the Bikerman model to include specific ion sizes have been developed iteratively in Section 4, Section 5 and Section 6, preceded by derivations of classical PB in Section 2, and the original Bikerman model in Section 3. Finally, in the Discussion and Conclusions section a specific-ion-size Bikerman model is presented with a guarantee that: (1) it can approach Boltzmann distribution at diluteness; (2) it can reach the saturation limit as the reciprocal of specific ion size under extreme electrostatic conditions; (3) its entropy can be derived by a mean-field lattice gas model.

## 2. Classical Poisson-Boltzmann Model

Though the classical PB model is well known, we still derive the model here for review and comparison with its modified versions discussed later. Starting by stating the Helmholtz free energy, internal energy and entropy, we have:

(1) F=U−TS

(2) $$U=\int \left[-\frac{\varepsilon }{2}{\left|\nabla \phi \right|}^{2}+z_{p}ep\phi +z_{n}en\phi +q\phi +pW_{sol,p}+nW_{sol,n}\right]dV,$$

(3) $$-TS=\int k_{B}T\left[p\;\log\frac{p}{c_{0}}-p+n\;\log\frac{n}{c_{0}}-n\right]dV,$$

where *F* is Helmholtz free energy; *U* is internal energy; *T* is temperature; *S* is entropy; ϕ is electric potential. *p*, *n* denote cation/anion concentrations, and $z_{p}$, $z_{n}$ denote their valence, respectively. *e* denotes elementary charge. *q* denotes permanent charge. Permittivity $\varepsilon =\varepsilon_{0}\varepsilon_{r}$ with $\varepsilon_{0}$ being the permittivity for vacuum and $\varepsilon_{r}$ being the relative permittivity or dielectric constant. $c_{0}$ is some reference concentration such as bulk concentration of electrolyte. $W_{sol,p}$ and $W_{sol,n}$ denote the solvation energies for cations and anions, respectively. Although the traditional PB model generally does not include solvation energy in the expression, it is important when modeling some electrolyte systems involving hydration/dehydration of ions and is therefore it is explicitly included in the energy here. Based on the Born model, the solvation energies for cations and anions are:

(4) $$W_{sol,i}=\frac{z_{i}^{2}e^{2}}{8\pi \varepsilon_{0}r_{i}}\left(\frac{1}{\varepsilon_{r}\left(x\right)}-1\right),\;i=p,n.$$

Differentiation of *F* with respect to ϕ gives the Poisson equation:

(5) $$-\nabla \cdot \left(\varepsilon \left(x\right)\nabla \phi \right)=z_{p}ep+z_{n}en+q$$

By doing the differentiation of *F* with respect to *p* and *n*, we obtain the chemical potentials for *p* and *n*, respectively:

(6) $$\frac{\partial F}{\partial p}=\mu_{p}=z_{p}e\phi +k_{B}T\;\log\frac{p}{c_{0}}+W_{sol,p},$$

(7) $$\frac{\partial F}{\partial n}=\mu_{n}=z_{n}e\phi +k_{B}T\;\log\frac{n}{c_{0}}+W_{sol,n}$$

At equilibrium, the chemical potential is uniform everywhere and therefore the local chemical potential must be equal to its bulk value, which is usually known:

(8) $$\mu_{p}=\mu_{p,b},\;\mu_{n}=\mu_{n,b},$$

with the bulk chemical potential for cations and anions:

(9) $$\mu_{p,b}=k_{B}T\;\log\left(\frac{p_{b}}{c_{0}}\right)+W_{sol,p,b},\;\;\mu_{n,b}=k_{B}T\;\log\left(\frac{n_{b}}{c_{0}}\right)+W_{sol,n,b},$$

where the subscript *b* denotes the bulk situation. Equations (8) and (9) can be solved solve for *p* and *n*:

(10) $$p=p_{b}e^{-\beta z_{p}e\phi }e^{-\beta \Delta W_{sol,p}}=p_{b}e^{-\beta E_{p}},\;n=n_{b}e^{-\beta z_{n}e\phi }e^{-\beta \Delta W_{sol,n}}=n_{b}e^{-\beta E_{n}},$$

where $\beta =1/k_{B}T$, $E_{p}=z_{p}e\phi +\Delta W_{sol,p},$ $E_{n}=z_{n}e\phi +\Delta W_{sol,n}$, and:

(11) $$\Delta W_{sol,i}=W_{sol,i}-W_{sol,i,b}=\frac{z_{i}^{2}e^{2}}{8\pi \varepsilon_{0}r_{i}}\left(\frac{1}{\varepsilon_{r}\left(x\right)}-\frac{1}{\varepsilon_{r,b}}\right),\;i=p,n.$$

From (10), as ϕ→−∞, we obtain p→∞, n→0. Likewise, as ϕ→∞, we obtain p→0, n→∞. These unrealistic infinite concentrations for *p* and *n* are mainly because ions are treated as particles without size in the classical PB model. This pitfall has motivated modifications of the classical PB/PNP model to account for the finite-size effect, or so-called steric effect, of ions. In reality, the limit of p should be at most $1/v_{p}$, where $v_{p}$ is the particle volume of p. This can be derived by considering a volume V fully occupied by cation *p* only, with the number of cation particles being $N_{p}$, and then:

(12) $$p_{max}=\frac{N_{p}}{V}=\frac{N_{p}}{N_{p}v_{p}}=\frac{1}{v_{p}}.$$

Likewise, the limit of n is at most $1/v_{n}$, where $v_{n}$ is the particle volume of n. 

Substituting (10) into (4), and we obtain the classical PB equation:

(13) $$-\nabla \cdot \left(\varepsilon \left(x\right)\nabla \phi \right)=z_{p}ep_{b}e^{-\beta E_{p}}+z_{n}en_{b}e^{-\beta E_{n}}+q.$$

For *z*:*z* electrolyte without considering solvation energy, the equation above reduces to:

(14) $$\nabla \cdot \left(\varepsilon \left(x\right)\nabla \phi \right)=2zec_{b}sinh\left(\beta ze\phi \right)-q,$$

with $c_{b}=p_{b}=n_{b}.$

## 3. Bikerman Model

As stated earlier, the Bikerman model [10] has been a popular steric-effect model due to its easiness of application and qualitatively good agreement with experimental data. It modifies the free energy of the classical PB (1)–(3) by adding a solvent entropy term. This term also partially represents the excessive energy accounting for overcrowding of ions and solvent molecules in localized hard-sphere models based on DFT [5,6,7,8]. The free energy in the Bikerman model treats all species of ions and solvent molecules with an identical size, and is stated as follows:

(15) F=U−TS,

(16) $$U=\int \left[-\frac{\varepsilon }{2}{\left|\nabla \phi \right|}^{2}+z_{p}ep\phi +z_{n}en\phi +q\phi +pW_{sol,p}+nW_{sol,n}\right]dV,$$

(17) $$-TS=\int k_{B}T\left[p\;\log\left(pv\right)-p+n\;\log\left(nv\right)-n+w\;\log\left(wv\right)-w\right]dV,$$

where w is concentration of solvent (such as water); *v* is the universal particle volume. Why are all solute and solvent particles treated as having the same size? Why are specific sizes of ions and solvent molecules not used here? This was not explained in the original model [10,11], and the justification of using identical size for all species particles will be addressed later in Section 5. 

If we assume that, besides occupation of ions, the rest of space is occupied by solvent molecules (which can be taken as water here). Then:

(18) $$w=\frac{N_{w}}{V}=\frac{N_{w}v}{Vv}=\frac{V-N_{p}v-N_{n}v}{Vv}=\frac{1}{v}\left(1-pv-nv\right),$$

where *V* is the whole volume of electrolyte; $N_{p},\;N_{n},\;N_{w}$ are number of cation, anion and solvent particles in an electrolyte with volume *V*, respectively. Equation (18) can then be rewritten as:

(19) wv+pv+nv=1,

which simply means the sum of volume fractions of water, cation and anion is one. Note that here we assume that, besides occupation of ions, the rest of space is occupied by water. 

Substituting (19) into (17) we can obtain:

(20) $$-TS=\int k_{B}T\left[p\;\log\left(pv\right)-p+n\;\log\left(nv\right)-n+\frac{1}{v}\left(1-pv-nv\right)\;\log\left(1-pv-nv\right)-\frac{1}{v}\left(1-pv-nv\right)\right]dV.$$

Differentiation of *F* with respect to ϕ again gives the Poisson equation:

(21) $$-\nabla \cdot \left(\varepsilon \left(x\right)\nabla \phi \right)=z_{p}ep+z_{n}en+q.$$

By doing the derivation of *F* with respect to *p* and *n*, we obtain the chemical potentials for *p* and *n*, respectively:

(22) $$\frac{\partial F}{\partial p}=\mu_{p}=z_{p}e\phi +k_{B}T\left[log\left(pv\right)-log\left(1-pv-nv\right)\right]+W_{sol,p},$$

(23) $$\frac{\partial F}{\partial n}=\mu_{n}=z_{n}e\phi +k_{B}T\left[log\left(nv\right)-log\left(1-pv-nv\right)\right]+W_{sol,p}.$$

At equilibrium, the chemical potential is uniform everywhere and therefore the local chemical potential must be equal to its bulk value, which is usually known:

(24) $$\mu_{p}=\mu_{p,b},\;\;\;\;\mu_{n}=\mu_{n,b},$$

with:

(25) $$\mu_{p,b}=k_{B}T\left[\log\left(p_{b}v\right)-\log\left(1-p_{b}v-n_{b}v\right)\right]+W_{sol,p,b},$$

(26) $$\mu_{n,b}=k_{B}T\left[\log\left(n_{b}v\right)-\log\left(1-p_{b}v-n_{b}v\right)\right]+W_{sol,n,b}.$$

By substituting (22), (23), (25) and (26) into (24), we can relate the local ion-to-solvent volume fraction ratios (denoted as $\gamma_{i},\;i=p,n.$) to their counterparts in bulk solution in a Boltzmann manner for *p* and *n*, respectively:

(27) $$\gamma_{p}=\frac{pv}{1-pv-nv}=\frac{p_{b}v}{1-p_{b}v-n_{b}v}e^{-\beta E_{p}},$$

(28) $$\gamma_{n}=\frac{nv}{1-pv-nv}=\frac{n_{b}v}{1-p_{b}v-n_{b}v}e^{-\beta E_{n}}.$$

Summation of (27) and (28) gives the solute-to-solvent volume fraction ratio as:

$$\frac{pv+nv}{1-pv-nv}=\gamma_{p}+\gamma_{n},$$

and we can further obtain the solute volume fraction:

(29) $$pv+nv=\frac{\gamma_{p}+\gamma_{n}}{1+\gamma_{p}+\gamma_{n}}.$$

From (29) and (19), we know then volume fraction for *p*, *n* and *w*, respectively.

(30) $$pv=\frac{\gamma_{p}}{1+\gamma_{p}+\gamma_{n}},\;nv=\frac{\gamma_{n}}{1+\gamma_{p}+\gamma_{n}},\;wv=\frac{1}{1+\gamma_{p}+\gamma_{n}},\;$$

which further gives *p* and *n* in terms of their bulk values $p_{b}$, $n_{b}$, identical particle size *v*, and local energy $E_{p}$, $E_{n}$:

(31) $$p=\frac{p_{b}e^{-\beta E_{p}}}{\left(1-p_{b}v-n_{b}v\right)+p_{b}ve^{-\beta E_{p}}+n_{b}ve^{-\beta E_{n}}},\;\;n=\frac{n_{b}e^{-\beta E_{n}}}{\left(1-p_{b}v-n_{b}v\right)+p_{b}ve^{-\beta E_{p}}+n_{b}ve^{-\beta E_{n}}}$$

Equations (27) and (28) can be re-arranged to obtain:

(32) $$p=p_{b}e^{-\beta (E_{p}+S^{trc})},\;\;n=n_{b}e^{-\beta (E_{n}+S^{trc})},$$

with ionic steric potential $S^{trc}$ expressed as:

(33) $$S^{trc}=k_{B}T\;\log\left(\frac{1-p_{b}v-n_{b}v}{1-pv-nv}\right).$$

The steric potential $S^{trc}$, first described in [16,17,18,19], characterizes the crowding of ions and their finite-size effect by a bulk-to-local water fraction ratio. Larger local ion concentrations would have a larger steric potential.

Also, by letting 1−pv−nv=wv, and $1-p_{b}v-n_{b}v=w_{b}v$, Equations (27) and (28) can be simplified as:

(34) $$\frac{pv}{wv}=\frac{p_{b}v}{w_{b}v}e^{-\beta E_{p}}=\gamma_{p},$$

(35) $$\frac{nv}{wv}=\frac{n_{b}v}{w_{b}v}e^{-\beta E_{n}}=\gamma_{n}.$$

Therefore, by (30), we obtain:

(36) $$z_{p}pv+z_{n}nv=\frac{z_{p}\frac{p_{b}v}{w_{b}v}e^{-\beta E_{p}}+z_{n}\frac{n_{b}v}{w_{b}v}e^{-\beta E_{n}}}{1+\frac{p_{b}v}{w_{b}v}e^{-\beta E_{p}}+\frac{n_{b}v}{w_{b}v}e^{-\beta E_{n}}}=\frac{z_{p}p_{b}ve^{-\beta E_{p}}+z_{n}n_{b}ve^{-\beta E_{n}}}{w_{b}v+p_{b}ve^{-\beta E_{p}}+n_{b}ve^{-\beta E_{n}}}.$$

Since $\frac{p_{b}}{n_{b}}=\frac{-z_{n}}{z_{p}}$ due to electric neutrality in bulk conditions, therefore:

(37) $$p_{b}v=\frac{-z_{n}\mu }{z_{p}-z_{n}}v,\;n_{b}v=\frac{z_{p}\mu }{z_{p}-z_{n}}v,$$

where $\mu =p_{b}+n_{b}$. Also:

(38) $$w_{b}v=1-p_{b}v-n_{b}v=1-\mu v.$$

then (36) becomes:

(39) $$z_{p}p+z_{n}n=\frac{z_{p}z_{n}\mu \left(-e^{-\beta E_{p}}+e^{-\beta E_{n}}\right)}{\left(1-\mu v\right)\left(z_{p}-z_{n}\right)+\mu v\left(z_{p}e^{-\beta E_{n}}-z_{n}e^{-\beta E_{p}}\right)}.$$

Substituting (39) into (21), we obtain the Bikerman-PB equation:

(40) $$-\nabla \cdot \left(\varepsilon \left(x\right)\nabla \phi \right)=\frac{z_{p}z_{n}e\mu \left(-e^{-\beta E_{p}}+e^{-\beta E_{n}}\right)}{\left(1-\mu v\right)\left(z_{p}-z_{n}\right)+\mu v\left(z_{p}e^{-\beta E_{n}}-z_{n}e^{-\beta E_{p}}\right)}+q.$$

For *z*:*z* electrolyte without considering the solvation energy, the equation above becomes:

(41) $$\nabla \cdot \left(\varepsilon \left(x\right)\nabla \phi \right)=\frac{2zec_{0}sinh\left(\beta ze\phi \right)}{1+2rsinh^{2}\left(\frac{\beta ze\phi }{2}\right)}-q,$$

as shown in [11] with $c_{0}=p_{b}=n_{b},$ $r=\mu v=2c_{0}v$.

Two important criteria need to be checked for all modified PB/PNP models accounting for steric effects:

CRITERION I: When ion concentrations *p* and *n* are dilute, will they follow the classical Boltzmann distribution? 

CRITERION II: As ϕ→∓∞, will *p* and *n* approach their saturation limits $\frac{1}{v_{p}}$ and $\frac{1}{v_{n}}$, respectively? 

For CRITERION I when *p* and *n* are dilute here, it means their volume fractions are negligible, and therefore 1−pv−nv≈1, and $1-p_{b}v-n_{b}v\approx 1$. Steric potential term $S^{trc}$ then vanishes, and by (32) $p=p_{b}e^{-\beta E_{p}}$, and $n=n_{b}e^{-\beta E_{n}}$, which follows the Boltzmann distribution.

For CRITERION II, let us consider ϕ→−∞ first, and ϕ→∞ can be derived similarly. As ϕ→−∞, $\gamma_{p}\to \infty$, and $\gamma_{n}\to 0$. Therefore, pv→1, and nv→0 by (30), which further means $p\to \frac{1}{v_{p}}=\frac{1}{v}$, and n→0. Likewise, as ϕ→∞, we can get $n\to \frac{1}{v_{n}}=\frac{1}{v}$, and p→0.

## 4. The Bikerman Model with Specific Ion Sizes

The shortcoming of the Bikerman model is the usage of a universal particle size, denoted by *v*, for cations, anions and solvents. Using specific ion and solvent sizes would be closer to reality. Taking NaCl solution as an example, the spherical diameters for Cl^−^, Na^+^ and water are $D_{Cl^{-}}=3.62Å$, $D_{Na^{+}}=2.04Å$, and $D_{w}=2.08Å$, and then the particle volume ratio is $v_{Na^{+}}:v_{Cl^{-}}:v_{w}=1:5.59:1.06$, in which using universal particle volume would be far from reality in the case of high ion concentrations. In appearance, it seems, and many researchers did, we can just simply modify the model to include specific ion sizes by changing pv and $p_{b}v$ to $pv_{p}$ and $p_{b}v_{p}$; similarly, nv and $n_{b}v$ to $nv_{n}$ and $n_{b}v_{n}$ for (22) to (41). With this straightforward extension, we obtain *p*, *n* as:

$$p=\frac{p_{b}e^{-\beta E_{p}}}{\left(1-p_{b}v_{p}-n_{b}v_{n}\right)+p_{b}v_{p}e^{-\beta E_{p}}+n_{b}v_{n}e^{-\beta E_{n}}},$$

(42) $$n=\frac{n_{b}e^{-\beta E_{n}}}{\left(1-p_{b}v_{p}-n_{b}v_{n}\right)+p_{b}v_{p}e^{-\beta E_{p}}+n_{b}v_{n}e^{-\beta E_{n}}}$$

and the specific-ion-size Bikerman-PB equation:

(43) $$-\nabla \cdot \left(\varepsilon \left(x\right)\nabla \phi \right)=\frac{e\left(z_{p}p_{b}e^{-\beta E_{p}}+z_{n}n_{b}e^{-\beta E_{n}}\right)}{1-\left(p_{b}v_{p}+n_{b}v_{n}\right)+\left(p_{b}+n_{b}\right)\left(z_{p}v_{n}e^{-\beta E_{n}}-z_{n}v_{p}e^{-\beta E_{p}}\right)/\left(z_{p}-z_{n}\right)}+q$$

For *z*:*z* electrolyte without considering solvation energy, the equation above becomes:

(44) $$\nabla \cdot \left(\varepsilon \left(x\right)\nabla \phi \right)=\frac{2zec_{0}sinh\left(\beta ze\phi \right)}{1-c_{0}\left(v_{p}+v_{n}\right)+c_{0}\left(v_{n}e^{\beta ze\phi }+v_{p}e^{-\beta ze\phi }\right)}-q.$$

Let us denote (42) as the specific-ion-size Bikerman model 1 (SISBM1) for convenience of notation. However, we can not find an energy functional like (15)–(17) to support this naive extension, which means chemical potentials (22) and (23) with universal particle size replaced by specific ion sizes cannot be derived. The correct specific-ion-size energy functional and chemical potentials should be derived as follows:

(45) F=U−TS, 

(46) $$U=\int \left[-\frac{\varepsilon }{2}{\left|\nabla \phi \right|}^{2}+z_{p}ep\phi +z_{n}en\phi +q\phi +pW_{sol,p}+nW_{sol,n}\right]dV,\;$$

(47) $$-TS=\int k_{B}T\left[p\;\log\left(pv_{p}\right)-p+n\;\log\left(nv_{n}\right)-n+w\;\log\left(wv_{w}\right)-w\right]dV.$$

By $wv_{w}=1-pv_{p}-nv_{n},$ (47) can be rewritten as:

(48) $$-TS=\int k_{B}T\left[p\;\log\left(pv_{p}\right)-p+n\;\log\left(nv_{n}\right)-n+\frac{1}{v_{w}}\left(1-pv_{p}-nv_{n}\right)\;\log\left(1-pv_{p}-nv_{n}\right)-\frac{1}{v_{w}}\left(1-pv_{p}-nv_{n}\right)\right]dV.$$

Differentiation of *F* with respect to ϕ again gives the Poisson equation:

(49) $$-\nabla \cdot \left(\varepsilon \left(x\right)\nabla \phi \right)=z_{p}ep+z_{n}en+q.$$

By doing the differentiation of *F* with respect to *p* and *n*, we can obtain the chemical potentials for cations and anions, respectively:

(50) $$\mu_{p}=z_{p}e\phi +k_{B}T\left[\log\left(pv_{p}\right)-\log{\left(1-pv_{p}-nv_{n}\right)}^{k_{p}}\right]+W_{sol,p}$$

(51) $$\mu_{n}=z_{n}e\phi +k_{B}T\left[\log\left(nv_{n}\right)-\log{\left(1-pv_{p}-nv_{n}\right)}^{k_{n}}\right]+W_{sol,n}$$

where $k_{p}=\frac{v_{p}}{v_{w}},$ $k_{n}=\frac{v_{n}}{v_{w}}$. 

At equilibrium, the chemical potential is uniform everywhere and therefore the local chemical potential must be equal to its bulk value, which is usually known:

(52) $$\mu_{p}=\mu_{p,b},\;\;\mu_{n}=\mu_{n,b},$$

with:

(53) $$\mu_{p,b}=k_{B}T\left[\log\left(p_{b}v_{p}\right)-\log{\left(1-p_{b}v_{p}-n_{b}v_{n}\right)}^{k_{p}}\right]+W_{sol,p,b},\;$$

(54) $$\mu_{n,b}=k_{B}T\left[\log\left(n_{b}v_{n}\right)-\log{\left(1-p_{b}v_{p}-n_{b}v_{n}\right)}^{k_{n}}\right]+W_{sol,n,b}.$$

To solve *p* and *n* from (52), there is no closed form solution like (31) for *p* and *n* due to the nonlinearity, unless some simplified case such as $k_{p}=k_{n}=1$ is considered, which is actually reduced to the original Bikerman model with $v_{p}=v_{n}=v_{w}=v$. Like (32), *p* and *n* at most can be expressed as:

(55) $$p=p_{b}e^{-\beta (E_{p}+k_{p}S^{trc})},\;\;n=n_{b}e^{-\beta (E_{n}+k_{n}S^{trc})},$$

with the steric potential $S^{trc}$ being modified from (33) to include specific ion sizes:

(56) $$S^{trc}=k_{B}T\;\log\left(\frac{1-p_{b}v_{p}-n_{b}v_{n}}{1-pv_{p}-nv_{n}}\right)$$

Let us denote (55) as the specific-ion-size Bikerman model 2 (SISBM2) for convenience. Note that a similar model was also obtained in [18,19] without a rigorous derivation.

Again, we need to check criteria I and II for this specific-ion-size model. For CRITERION I, when *p* and *n* are dilute, it again means $1-pv_{p}-nv_{n}\approx 1,$ and $1-p_{b}v_{p}-n_{b}v_{n}\approx 1$. Therefore $S^{trc}\approx 0$ by (56), and then $p=p_{b}e^{-\beta E_{p}}$, and $n=n_{b}e^{-\beta E_{n}}$ by (55), which follows a classical Boltzmann distribution. For CRITERION II, as ϕ→−∞ in (50), $k_{B}T\left[\log\left(pv_{p}\right)-\log{\left(1-pv_{p}-nv_{n}\right)}^{k_{p}}\right]$ should approach +∞ for $\mu_{p}$ to be finite. This can only be achieved by n→0, and $p\to {\left(\frac{1}{v_{p}}\right)}^{-}$ (saturation). Applying the same reasoning for (51), as ϕ→∞, $k_{B}T\left[\log\left(nv_{n}\right)-\log{\left(1-pv_{p}-nv_{n}\right)}^{k_{n}}\right]$ should approach +∞ for $\mu_{n}$ to be finite. Then p→0, and $n\to {\left(\frac{1}{v_{n}}\right)}^{-}$ (saturation). This specific-ion-size model seems correct and reasonable so far, but actually there is a pitfall. That is its entropy formula (48) cannot be derived by the traditional mean-field lattice gas model. This will be explained in the next section.

## 5. Mixing Entropy Derivation Based on the Mean-Field Lattice Gas Model

**Figure 1:**
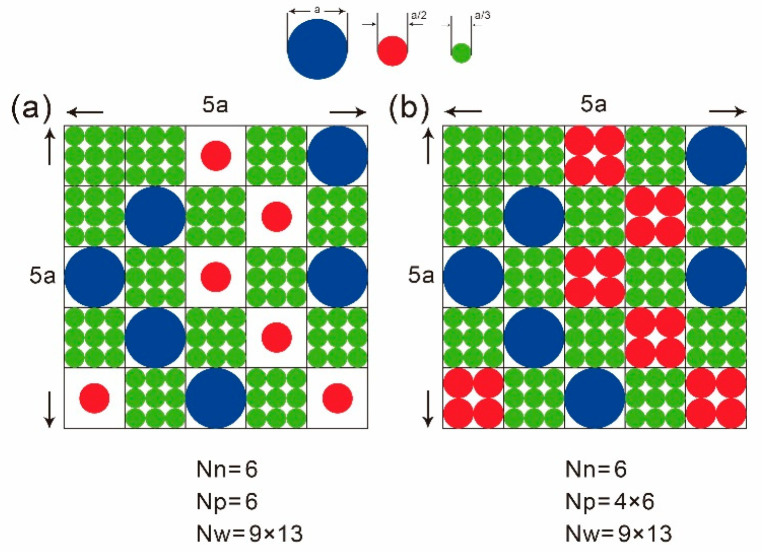
(**a**) Moderately concentrate situation with each solute/solvent particle only occupy one identical site. (**b**) Extremely concentrate situation with each identical site can be allowed to be occupied by multiple solute/solvent particles of the same species in order to increase packing efficiency in space.

In this section, we would like to derive the entropy in (20) by the traditional mean-field lattice gas model. Consider the entropy for an aqueous electrolyte system:

(57) $$TS=k_{B}T\;\log\;W,$$

where *W* is the number of microstates at equilibrium which possess a maximum number of microstates. Mixing entropy in electrolyte studies macrostates through spherical particles’ (solute and solvent) occupation of identical cubic sites is based on the mean-field lattice gas model. The necessity of using identical cubic sites provides a combinatorial basis when computing the maximum number of microstates. The most probable distribution of all solute (ions) and solvent particles, reaching maximum number of microstates for each species, is that each identical cubic site generally would be at most occupied by one solute/solvent particle as depicted in Figure 1a. This is based on the concept that the size of each species’ particle is infinitesimal or finite but dilute. When the actual size for each species’ particle is considered and an aqueous electrolyte is extremely concentrated as depicted in Figure 1b, the most probable distribution above may not be available. The situation in Figure 1b will be addressed in the next section. 

The entropy based on the most probable distribution of *K*-species solute (ions) and solvent (treated as K+1-th species) particles, under dilute situation, over a total of $N=\sum_{j=1}^{K+1}N_{j}$ available identical sites in a system is:

(58) $$\begin{array}{l} W\\ =\overset{K+1}{\underset{j=1}{\prod }}W_{j}\\ =\left(\begin{array}{c} N\\ N_{1} \end{array}\right)\left(\begin{array}{c} N-N_{1}\\ N_{2} \end{array}\right)\cdots \left(\begin{array}{c} N-N_{1}-N_{2}\cdots -N_{K-1}\\ N_{K} \end{array}\right)\left(\begin{array}{c} N-N_{1}-N_{2}\cdots -N_{K}\\ N_{K+1} \end{array}\right)\\ =\frac{N!}{N_{1}!\left(N-N_{1}\right)!}\frac{\left(N-N_{1}\right)!}{N_{2}!\left(N-N_{1}-N_{2}\right)!}\cdots \frac{\left(N-\sum_{j=1}^{K-1}N_{j}\right)!}{N_{K}!\left(N-\sum_{j=1}^{K}N_{j}\right)!}\frac{\left(N-\sum_{j=1}^{K}N_{j}\right)!}{N_{K+1}!}\\ =\frac{N!}{\left(\prod_{j=1}^{K}N_{j}!\right)N_{K+1}!}, \end{array}$$

where $N_{j},\;j=1,\cdots ,K,$ is the particle number of *j*-species ion. $N_{K+1}$ is the particle number of solvent, so the entropy becomes:

(59) $$TS=k_{B}T\;\log\frac{N!}{\left(\prod_{j=1}^{K}N_{j}!\right)N_{K+1}!}.$$

Using the Stirling formula logM!≈MlogM−M with M≫1, we can rewrite the entropy as:

(60) $$\begin{array}{cc} TS & =k_{B}T\;\left[N\log N-N-\overset{K}{\underset{j=1}{\sum }}N_{j}\log N_{j}+\overset{K}{\underset{j=1}{\sum }}N_{j}-N_{K+1}\log N_{K+1}+N_{K+1}\right]\\ & =k_{B}T\left[N\log N-\overset{K}{\underset{j=1}{\sum }}N_{j}\log N_{j}-N_{K+1}\log N_{K+1}\right]\\ & =k_{B}T\left[N\log N-\overset{K}{\underset{j=1}{\sum }}N_{j}\log N_{j}-\left(N-\overset{K}{\underset{j=1}{\sum }}N_{j}\right)\log\left(N-\overset{K}{\underset{j=1}{\sum }}N_{j}\right)\right]\\ & =k_{B}T\left[N\log\frac{N}{N-\sum_{j=1}^{K}N_{j}}-\overset{K}{\underset{j=1}{\sum }}N_{j}\log\frac{N_{j}}{N-\sum_{j=1}^{K}N_{j}}\right] \end{array}$$

using the following relations:

(61) $$V=Nv_{s},\;or\;\frac{N}{V}=\frac{1}{v_{s}},$$

(62) $$\frac{N_{j}}{V}=c_{j},$$

(63) $$\frac{N_{j}}{N}=\frac{N_{j}v_{s}}{Nv_{s}}=\frac{N_{j}v_{s}}{V}=c_{j}v_{s},$$

where $c_{j}$ is the concentration of *j*-species particle; *V* is the volume of system; $v_{s}$ is the volume of an identical cubic site that composes the volume of system. It is naturally requested that $v_{s}\geq max_{1\leq j\leq K+1}v_{j}$, where $v_{j}$ is the particle volume of *j*-species particle. Usually $v_{s}=max_{1\leq j\leq K+1}v_{j}$ in aqueous electrolyte system, where solute and solvent particles are generally crowded. 

Applying (61)–(63) to (60), the entropy density can be expressed as:

(64) $$\frac{TS}{V}=k_{B}T\left[\frac{1}{v_{s}}\log\frac{1}{1-\sum_{j=1}^{K}c_{j}v_{s}}-\overset{K}{\underset{j=1}{\sum }}c_{j}\log\frac{c_{j}v_{s}}{1-\sum_{j=1}^{K}c_{j}v_{s}}\right].$$

For a binary electrolyte, (64) can be expressed as:

(65) $$TS=\int k_{B}T\left[\frac{1}{v_{s}}\log\frac{1}{1-pv_{s}-nv_{s}}-p\;\log\frac{pv_{s}}{1-pv_{s}-nv_{s}}-n\;\log\frac{nv_{s}}{1-pv_{s}-nv_{s}}\right]dV,$$

or:

(66) $$-TS=\int k_{B}T\left[p\;\log\left(pv_{s}\right)+n\;\log\left(nv_{s}\right)+\frac{1}{v_{s}}\left(1-pv_{s}-nv_{s}\right)\log\left(1-pv_{s}-nv_{s}\right)\right]dV,$$

which, without loss of generality, can be augmented as:

(67) $$-TS=\int k_{B}T\left[p\;\log\left(pv_{s}\right)-p+n\;\log\left(nv_{s}\right)-n+\frac{1}{v_{s}}\left(1-pv_{s}-nv_{s}\right)\log\left(1-pv_{s}-nv_{s}\right)-\frac{1}{v_{s}}\left(1-pv_{s}-nv_{s}\right)\right]dV.\;$$

Equation (67) is exactly the same as (20) with:

(68) $$v=v_{s}=max\left\{v_{p},v_{n},v_{w}\right\}.$$

This means the universal particle volume v in the Bikerman model is actually the volume of an identical occupation site $v_{s}$, which is limited from below by the largest particle size among all solute and solvent particles. The original Bikerman model has long suffered criticism for assuming all ions have the same size instead of using specific ion sizes in the model. The above reasoning explains why specific ion size information is left out mainly due to the need for all cubic sites to be identical in order to support the combinatorial basis demanded by the mean-field lattice gas model. Actually, information of specific ion sizes is still carried but only implicitly as shown in (68). Researchers may prefer to use SISBM2 as illustrated in Section 4, but actually its entropy formula (48) cannot be derived by the mean-field lattice gas model described above. Note that usually solute and solvent particles are treated as spheres in modeling. If $a_{p},a_{n},a_{w}$ are diameters for *p*, *n*, and *w*, respectively and their maximum is $a_{n}$ for example, then $v=v_{s}=a_{n}^{3}$ not $\frac{4\pi }{3}{\left(\frac{a_{n}}{2}\right)}^{3}$ since the identical occupation is cubic. This is why $a^{3}$, instead of $\frac{4\pi }{3}{\left(\frac{a}{2}\right)}^{3}$, used in [10,11].

In CRITERION II described above, as ϕ→∓∞, *p* and *n* should approach their saturation limits $\frac{1}{v_{p}}$ and $\frac{1}{v_{n}}$, respectively. Here, this would be changed to approach $\frac{1}{v_{s}}$ instead of $\frac{1}{v_{p}}$ and $\frac{1}{v_{n}}$ respectively, although approaching $\frac{1}{v_{p}}$ and $\frac{1}{v_{n}}$ sounds more physically correct. This paradoxical conclusion is from entropy rigorously derived by the traditional mean-field lattice gas model based on combinatorics requiring identical occupation sites. Can this be fixed to resume the limit approach to $\frac{1}{v_{p}}$ and $\frac{1}{v_{n}}$ and still holding the ground of combinatorics at the same time? An attempt at this is discussed in the next section. 

## 6. Entropy Fixing for Electrolytes under Extreme Concentration Conditions

Here we hope to construct a steric PB model with entropy able to be derived by the mean-field lattice gas model, and at the same time showing physically correct saturation limits for ions as ϕ→∓∞. The mean-field lattice gas model is fixed here such that each identical cubic site is allowed to be occupied by more than one solute particle of the same species as illustrated in Figure 1b. Although this kind of distribution is no more a most probable distribution as stated earlier, it allows more efficient packing when space is extremely limited and size among species varies largely. Again, we consider the entropy for an aqueous electrolyte system:

(69) $$TS=k_{B}T\;\log W,$$

Let ${\tilde{N}}_{j},\;j=1,\cdots ,K+1,$ be the particle number of species *j* and $N_{j},\;j=1,\cdots ,K+1,$ the number of identical sites occupied by *j*-species particles with ${\tilde{N}}_{j}\geq N_{j}$. This means that in an extremely concentrated situation an identical site can be occupied by more than one particle of the same species. If an identical cubic site, on average, can allow $r_{j}$ *j*-species particles to occupy it, we can then relate ${\tilde{N}}_{j}$ and $N_{j}$ by ${\tilde{N}}_{j}=N_{j}r_{j}$, or equivalently $v_{s}=r_{j}v_{j}$. Again, $v_{j}$ is the particle volume of species *j*. $v_{s}$ is the volume of an identical cubic site with $v_{s}=max_{1\leq j\leq K+1}v_{j}$. The entropy based on the most probable distribution of all ‘grouped’ species particles over a total of $N=\sum_{j=1}^{K+1}N_{j}$ available identical sites in a system is:

(70) $$W=\overset{K+1}{\underset{j=1}{\prod }}W_{j}=\frac{N!}{N_{1}!\left(N-N_{1}\right)!}\frac{\left(N-N_{1}\right)!}{N_{2}!\left(N-N_{1}-N_{2}\right)!}\cdots \frac{\left(N-\sum_{j=1}^{K-1}N_{j}\right)!}{N_{K}!\left(N-\sum_{j=1}^{K}N_{j}\right)!}\frac{\left(N-\sum_{j=1}^{K}N_{j}\right)!}{N_{K+1}!}=\frac{N!}{\left(\prod_{j=1}^{K}N_{j}!\right)N_{K+1}!}.$$

Note that, after all the ions (in group) are distributed, there are $N-\sum_{j=1}^{K}N_{j}=N_{K+1}$ sites that will be filled by solvent molecules in group, so the entropy becomes:

(71) $$TS=k_{B}T\;\log\frac{N!}{\left(\prod_{j=1}^{K}N_{j}!\right)N_{K+1}!}.$$

Using the Stirling formula logM!≈MlogM−M with M≫1, we can rewrite the entropy as:

(72) $$\begin{array}{c} TS=k_{B}T\;[N\log N-N-\sum_{j=1}^{K}N_{j}\log N_{j}+\sum_{j=1}^{K}N_{j}-\left(N-\sum_{j=1}^{K}N_{j}\right)\log\left(N-\sum_{j=1}^{K}N_{j}\right)\\ +\left(N-\sum_{j=1}^{K}N_{j}\right)]=k_{B}T\left[N\log\frac{N}{N-\sum_{j=1}^{K}N_{j}}-\sum_{j=1}^{K}N_{j}\log\frac{N_{j}}{N-\sum_{j=1}^{K}N_{j}}\right], \end{array}$$

Using the following relations:

(73) $$V=Nv_{s},\;\mathrm{or}\;\frac{N}{V}=\frac{1}{v_{s}},$$

(74) $$\frac{N_{j}}{V}=\frac{\frac{{\tilde{N}}_{j}}{r_{j}}\;}{V}=\frac{c_{j}}{r_{j}},$$

(75) $$\frac{N_{j}}{N}=\frac{N_{j}v_{s}}{Nv_{s}}=\frac{N_{j}v_{s}}{V}=\frac{c_{j}}{r_{j}}r_{j}v_{j}=c_{j}v_{j},$$

where $c_{j}$ is the concentration of species *j*; *V* is the volume of the system. 

The entropy per unit volume can be expressed as:

(76) $$\frac{TS}{V}=k_{B}T\left[\frac{1}{v_{s}}\log\frac{1}{1-\sum_{j=1}^{K}c_{j}v_{j}}-\overset{K}{\underset{j=1}{\sum }}\frac{c_{j}}{r_{j}}\log\frac{c_{j}v_{j}}{1-\sum_{j=1}^{K}c_{j}v_{j}}\right].$$

Compared with (64), specific ion sizes can now appear explicitly in the entropy formula (76). For a binary electrolyte:

(77) $$TS=\int k_{B}T\left[\frac{1}{v_{s}}\log\frac{1}{1-pv_{p}-nv_{n}}-\frac{p}{r_{p}}\log\frac{pv_{p}}{1-pv_{p}-nv_{n}}-\frac{n}{r_{n}}\log\frac{nv_{n}}{1-pv_{p}-nv_{n}}\right]dV,$$

or:

(78) $$-TS=\int k_{B}T\left[\frac{p}{r_{p}}\log\left(pv_{p}\right)+\frac{n}{r_{n}}\log\left(nv_{n}\right)+\frac{1}{v_{s}}\left(1-pv_{p}-nv_{n}\right)\log\left(1-pv_{p}-nv_{n}\right)\right]dV,$$

which, without loss of generality, can be augmented as:

(79) $$-TS=\int k_{B}T\left[\frac{p}{r_{p}}\log\left(pv_{p}\right)-\frac{p}{r_{p}}+\frac{n}{r_{n}}\log\left(nv_{n}\right)-\frac{n}{r_{n}}+\frac{1}{v_{s}}\left(1-pv_{p}-nv_{n}\right)\log\left(1-pv_{p}-nv_{n}\right)-\frac{1}{v_{s}}\left(1-pv_{p}-nv_{n}\right)\right]dV.\;$$

by:

(80) $$\mu_{p}=\frac{\delta f}{\delta p}=z_{p}e\phi +W_{sol,p}+\frac{k_{B}T}{r_{p}}\;\log\frac{pv_{p}}{1-pv_{p}-nv_{n}},$$

(81) $$\mu_{n}=\frac{\delta f}{\delta n}=z_{n}e\phi +W_{sol,n}+\frac{k_{B}T}{r_{n}}\;\log\frac{nv_{n}}{1-pv_{p}-nv_{n}},$$

Again, at equilibrium, the chemical potential is uniform everywhere and therefore the local chemical potential must be equal to its bulk value, which is usually known:

(82) $$\mu_{p}=\mu_{p,b},\;\;\mu_{n}=\mu_{n,b}.$$

Usually bulk solutions are dilute and chemical potentials under that condition can be formulated following (80) and (81) with $r_{p}=r_{n}=1$:

(83) $$\mu_{p,b}=k_{B}T\;\log\frac{p_{b}v_{p}}{1-p_{b}v_{p}-n_{b}v_{n}}+W_{sol,p,b},$$

(84) $$\mu_{n,b}=k_{B}T\;\log\frac{n_{b}v_{n}}{1-p_{b}v_{p}-n_{b}v_{n}}+W_{sol,n,b}.$$

By denoting $\gamma_{p}=e^{\frac{\mu_{p,b}-z_{p}e\phi -W_{sol,p}}{k_{B}T/r_{p}}\;},\;\gamma_{n}=e^{\frac{\mu_{n,b}-z_{n}e\phi -W_{sol,n}}{k_{B}T/r_{n}}}$, 

(82) forms

(85) $$\frac{pv_{p}}{1-pv_{p}-nv_{n}}=\gamma_{p},\;\;\frac{nv_{n}}{1-pv_{p}-nv_{n}}=\gamma_{n},$$

and can solve for *p* and *n*:

(86) $$pv_{p}=\frac{\gamma_{p}}{1+\gamma_{p}+\gamma_{n}},\;nv_{n}=\frac{\gamma_{n}}{1+\gamma_{p}+\gamma_{n}},$$

or:

(87) $$pv_{p}=\frac{{\left(\frac{p_{b}v_{p}}{1-p_{b}v_{p}-n_{b}v_{n}}\right)}^{r_{p}}e^{-\beta_{p}E_{p}}}{1+{\left(\frac{p_{b}v_{p}}{1-p_{b}v_{p}-n_{b}v_{n}}\right)}^{r_{p}}e^{-\beta_{p}E_{p}}+{\left(\frac{n_{b}v_{n}}{1-p_{b}v_{p}-n_{b}v_{n}}\right)}^{r_{n}}e^{-\beta_{n}E_{n}}}$$

(88) $$nv_{n}=\frac{{\left(\frac{n_{b}v_{n}}{1-p_{b}v_{p}-n_{b}v_{n}}\right)}^{r_{n}}e^{-\beta_{n}E_{n}}}{1+{\left(\frac{p_{b}v_{p}}{1-p_{b}v_{p}-n_{b}v_{n}}\right)}^{r_{p}}e^{-\beta_{p}E_{p}}+{\left(\frac{n_{b}v_{n}}{1-p_{b}v_{p}-n_{b}v_{n}}\right)}^{r_{n}}e^{-\beta_{n}E_{n}}},$$

where $E_{p}=z_{p}e\phi +\Delta W_{sol,p},$ $E_{n}=z_{n}e\phi +\Delta W_{sol,n}$, $\beta_{p}={\left(k_{B}T/r_{p}\right)}^{-1}$, $\beta_{n}={\left(k_{B}T/r_{n}\right)}^{-1}$. Let us denote (87), (88) as the specific-ion-size Bikerman model 3 (SISBM3) for convenience.

Again, we need to check this new model with criteria I and II. For CRITERION II, we can easily deduce from (87) ϕ→−∞, n→0, $pv_{p}\to 1,$ $p\to \frac{1}{v_{p}}$ (saturation). Similarly, from (88), ϕ→∞, p→0, $nv_{n}\to 1$, $n\to \frac{1}{v_{n}}$ (saturation). There is no constraint like $p,n\to \frac{1}{v_{s}}$ as ϕ→∓∞ any more, and entropy here can be derived by mean-field lattice gas model. 

For CRITERION I, p and n will not approach a Boltzmann distribution $p_{b}e^{-\beta E_{p}}$ and $n_{b}e^{-\beta E_{n}}$ at diluteness unless $r_{p}=r_{n}=1$. This violation of the Boltzmann distribution at the dilution limit is because we allow multiple ions of the same species to occupy an identical cubic site. This failure and a possible cure will be discussed in next section.

## 7. Discussion and Conclusions

If we wish to obtain a model for electrolytes such that: (1) it can approach a Boltzmann distribution at diluteness; (2) it can reach the saturation limit as the reciprocal of specific ion size under extreme electrostatic conditions; (3) its entropy can be derived by a mean-field lattice gas model. The only options here is SISBM3 with $r_{p}=r_{n}=1$, since SISBM2 satisfies (1) and (2) but not (3). How can we justify $r_{p}=r_{n}=1$ for SISBM3 here? Interpreting all ion sizes as being about the same is certainly not acceptable. Remember SISBM3 is designed for extremely high ion concentrations motivated by the more efficient packing shown in Figure 1b. Actually for situations that would give rise to extremely high ion concentrations and make the steric effect not negligible, such as the Stern layer in the electric double layer (EDL) of a charged wall (discussed next) and the selectivity filter of a K channel [20], there would be ‘locally’ one species only, which is the counter-ion of the local electrostatic environment, since co-ions (and even water) would be totally expelled. Taking the K channel selectivity filter as an example, its extreme narrowness and the strong negative oxygen charges inside it would definitely justify only one species being inside the selectivity filter, which is definitely potassium. This implies $r_{p}$ to be 1 locally in the filter, and we can justify $r_{n}=1$ inside the filter as well since anions would be extremely dilute there due to strong electrostatic repulsion. For the rest of the K channel where ions are at most moderately concentrated, the steric effect is much less significant, and basically the original Bikerman model would be appropriate for it. Since SISBM3 with $r_{p}=r_{n}=1$ would be a very good approximation of the original Bikerman model under mild ion concentrations, we can use SISBM3 with $r_{p}=r_{n}=1$ globally for the whole K channel then. Notice, under $r_{p}=r_{n}=1$, SISBM3 is actually same as SISBM1, but with a rigorous derivation now. This model has been useful and proven to fit the experimental data quite well [16,17,18]. Although here we just discussed steric-effect modifications for PB, modifications for PNP can be likewise derived. 

**Figure 2:**
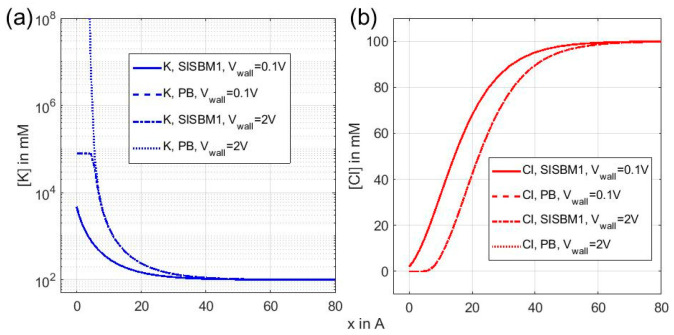
(**a**) K distributions in charged wall problem computed by SISBM1 and PB models under different wall voltages. (**b**) Cl distributions with conditions same as (**a**).

Here we compare SISBM1 and PB by computing ion distributions in a 1D charged wall problem. Many researchers have used this physical model to investigate the surface differential capacitance of electrodes adjacent to electrolyte solutions [12,13,14,15]. Here (14) for PB and (44) for SISBM1 were used to calculate the ion distributions of a binary KCl electrolyte solution without considering the solvation energy and permanent charges. The associated boundary conditions are $\phi \left(0\right)=V_{wall},$ and ϕ(∞)=0. The bulk concentration of KCl as x→∞ is set to be $c_{0}=100$ mM, and dielectric constant is set to 80 for the whole domain (0,∞). The Debye length, featuring the order of thickness of EDL, is $\lambda_{D}=\sqrt{\frac{\varepsilon_{0}\varepsilon_{r}k_{B}T}{c_{0}e^{2}}}=13.78Å$. The simulation result is shown in Figure 2 with Figure 2a being the distributions of $\left[K^{+}\right]$ for SISBM1 and PB under $V_{wall}=0.1$ V and 2 V. Figure 2b is the counterpart plot of Figure 2a for $\left[Cl^{-}\right]$. In Figure 2a, the $\left[K^{+}\right]$ distributions for SISBM1 and PB are very close to each other and almost indistinguishable in the graph at a weak wall voltage $V_{wall}=0.1$ V. When the wall voltage increases to $V_{wall}=2$ V, $\left[K^{+}\right]$ calculated by SISBM1 reaches its saturation limit $1/v_{K}=7.90\times 10^{4}$ mM right adjacent to the wall, but $\left[K^{+}\right]$ unrealistically increases beyond the saturation limit when computed by the PB model. The main effect of SISBM1 is to offer a saturation limit for counter-ions (K here) when electrostatic attraction from electrode is strong enough, while it is very close to the result of PB when the electrostatic attraction is weak. In Figure 2b, $\left[Cl^{-}\right]$ distributions calculated by SISBM1 and PB are very close to each other for both strong and weak wall voltages due to the diluteness caused by electrostatic repulsion to the co-ion (Cl here) of the electrode. Note that, corresponding to a saturation layer of $\left[K^{+}\right]$ adjacent to wall at $V_{wall}=2$ V (see Figure 2a), $\left[Cl^{-}\right]$ almost vanishes at that layer as well (see Figure 2b). This implies a total exclusion of Cl over there due to the saturation of K, and justifies the locally one-species argument above. If we use the original Bikerman model (41), in which ion sizes are universal, a similar saturating phenomenon for counter-ion concentration can still be obtained. However, specific ion sizes are particularly desired when electrolyte solutions are ternary, like a mixture of KCl and NaCl solutions since K and Na have different sizes, which would saturate at different limiting concentrations. These would be otherwise indistinguishable if using the original Bikerman model.

Above we assume the rest of space after the occupation of ions is exclusively occupied by solvent particles such as water. [9,10,11,12] have suggested that the rest of space should be occupied by solvent or void, so the K+1 species in (58) and (70) should be interpreted as solvent or void. This may make more sense. Taking the selectivity filter of a K channel as an example, more and more evidences have shown the selectivity filter of a K channel is exclusively occupied by potassium and voids, and water is not allowed there due to the strong solvation energy barrier. [9,10,11,12] even explicitly separate water and voids as two species in their modeling. However, that means the species transport equation (Nernst-Planck equation) of water needs to be modeled explicitly when constructing a PNP type model. This water equation is generally hard to model due to its physical complexity, especially for its electrostatic behavior since water is a dipole.
